# Evaluation of *SEPT2* and *SEPT4* transcript contents in spermatozoa from men with asthenozoospermia and teratozoospermia

**DOI:** 10.1002/hsr2.436

**Published:** 2021-11-23

**Authors:** Madiheh Mazaheri Moghaddam, Marziyeh Mazaheri Moghaddam, Mohammad Amini, Behzad Bahramzadeh, Amir Baghbanzadeh, Alireza Biglari, Ebrahim Sakhinia

**Affiliations:** ^1^ Department of Genetics and Molecular Medicine School of Medicine, Zanjan University of Medical Sciences (ZUMS) Zanjan Iran; ^2^ Department of Medical Genetics, Faculty of Medicine Tabriz University of Medical Sciences Tabriz Iran; ^3^ Immunology Research Center Tabriz University of Medical Sciences Tabriz Iran; ^4^ Al‐Zahra Hospital, Women's Reproductive Health Research Center Tabriz University of Medical Sciences Tabriz Iran

**Keywords:** asthenozoospermia, *SEPT2*, *SEPT4*, sperm annulus, teratozoospermia

## Abstract

**Background and aims:**

Motility and morphological defects of spermatozoa can cause male infertility. Sperm RNAs are related to sperm quality. They are considered to have clinical values as a biomarker for assessing sperm quality and fertility potential. The annulus, located in the mammalian sperm tail, is required for motility and terminal differentiation of the spermatozoa. SEPT2, 4, 6, 7, and 12 proteins are the main components of the annulus in the sperm tail. The study aimed to evaluate *SEPT2* and *SEPT4* mRNA contents in the spermatozoa of patients with asthenozoospermia and teratozoospermia.

**Methods:**

We evaluated transcript levels of *SEPT2* and *SEPT4* in the sperm samples of 20 asthenozoospermic, 20 teratozoospermic, and 20 normozoospermic samples using quantitative PCR.

**Results:**

The *SEPT2* transcript level was significantly decreased in the asthenozoospermia samples compared with the normal group (*P* = .013). However, *SEPT4* was not significantly different between these two groups. The transcript levels of *SEPT2* and *SEPT4* were not statistically different between teratozoospermic and normozoospermic groups.

**Conclusion:**

In conclusion, downregulation of *SEPT2* in patients with asthenozoospermia appears to be associated with poor sperm motility.

## 1 INTRODUCTION

Infertility is defined as the failure to achieve pregnancy after 1 year of regular unprotected intercourse.^1^ It affects approximately 15% of couples, and half of the cases are due to male factor infertility.^2^ Motility and morphological defects of spermatozoa are well documented to cause male infertility.^3^, ^4^ Evidence accumulated over the past decades has revealed that sperm RNAs are related to sperm quality.^5^ It is believed that mRNAs in ejaculated spermatozoa are remnants of transcripts synthesized during spermatogenesis, provide a historical record of spermatogenesis, and could be used to identify the genes responsible for sperm structure and function.^6^, ^7^, ^8^, ^9^, ^10^ Several transcripts are present in different amounts between fertile individuals and patients with asthenozoospermia and teratozoospermia. These findings might help to identify the pathogenic mechanisms involved in impaired motility and morphology of spermatozoa. Consequently, sperm mRNAs might be useful as potential biomarkers for assessing sperm quality and fertility potential.^8^, ^11^, ^12^, ^13^, ^14^, ^15^, ^16^, ^17^, ^18^, ^19^, ^20^

**FIGURE 1:**
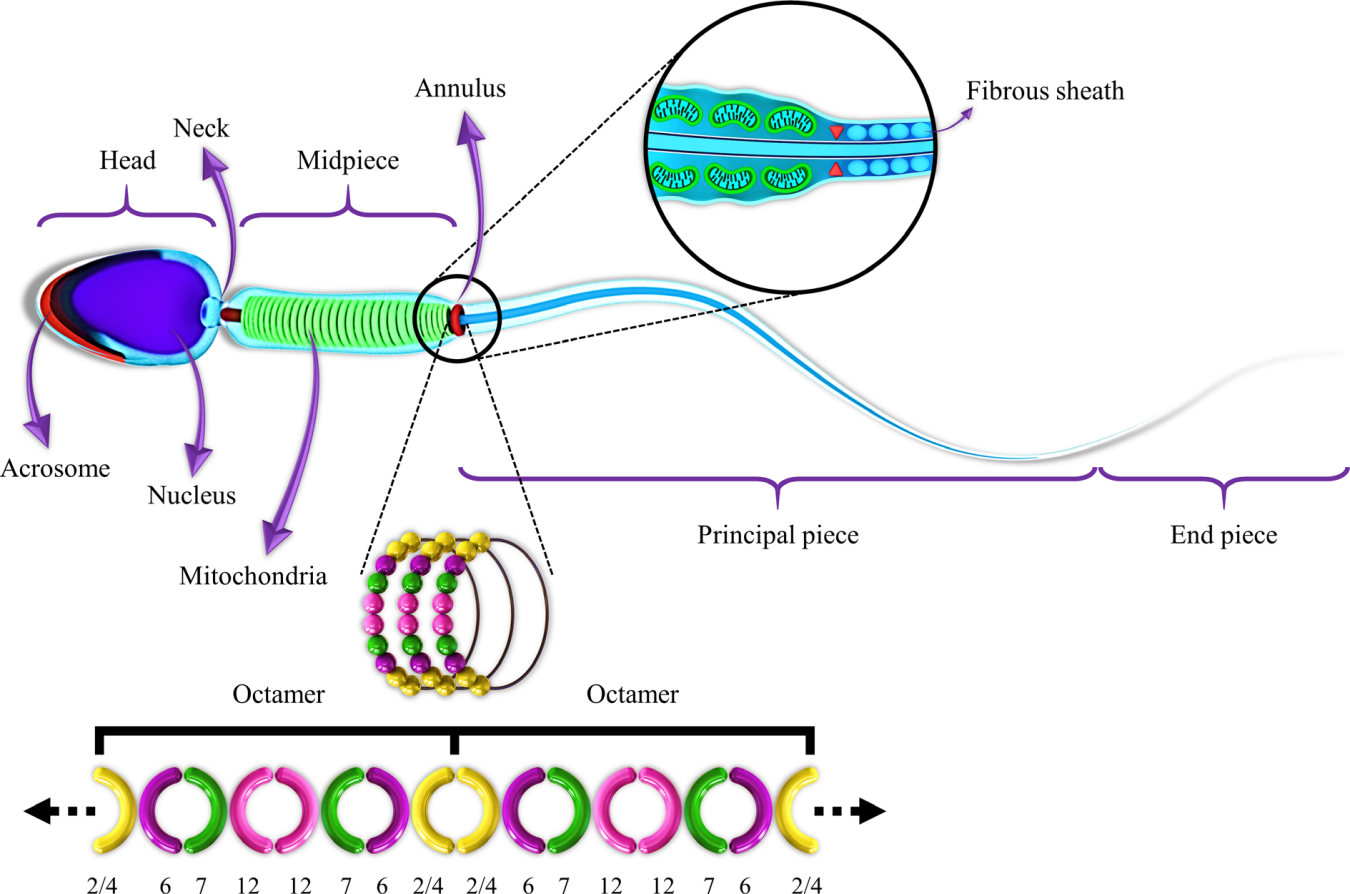
Schematic diagrams of normal sperm and formation of the annulus. The sperm cell contains a head and a tail. The sperm head and tail are connected by the neck. The tail or flagellum consists of three parts: the midpiece, principal piece, and end piece. The annulus is localized between the midpiece and the principal piece. Kuo et al. have revealed that SEPT2, 4, 6, 7, and 12 proteins are the annulus components forming 12‐7‐6‐2‐2‐6‐7‐12 or 12‐7‐6‐4‐4‐6‐7‐12 octamers.^26^ However, recent studies have revised the order of mammalian septin complexes and revealed the position of SEPT2 at the ends of the octamer.^27^, ^28^, ^29^ So the order suggested by Kuo et al probably should be inverted, and we might expect 2‐6‐7‐12‐12‐7‐6‐2 or 4‐6‐7‐12‐12‐7‐6‐4 positions in octamers. SEPT2 and SEPT4 occupy the same position in this complex. These octameric complexes form the ring structure of the septin in the annulus by end‐to‐end association.^26^ Autodesk 3ds Max was applied to draw this artwork

The annulus connects the midpiece and principal piece of the mammalian sperm tail. It acts as a diffusion barrier and is required for motility, cortical organization, and terminal differentiation of the spermatozoa.^21^, ^22^, ^23^, ^24^ However, the accurate mechanisms of the annulus biogenesis and function are unknown.^25^ SEPT2, 4, 6, 7, and 12 proteins are the main components of the annulus in the sperm tail (Figure 1).^26^ According to studies in humans and mice, *SEPT4* and *SEPT12* are required for sperm motility and structural integrity.^21^, ^30^ *SEPT4* has been identified to be correlated with motility and morphological defects of spermatozoa.^21^, ^22^, ^31^ However, the association between *SEPT4* transcript quantity and teratozoospermia has not been reported. Also, as we know, *SEPT2* has not been investigated in patients with asthenozoospermia and teratozoospermia by quantitative PCR (qPCR) thus far. Therefore, we evaluated *SEPT2* and *SEPT4* transcript contents in patients with asthenozoospermia and teratozoospermia by qPCR in this study.

## 2 METHODS

### 2.1 Sample collection and processing

This study was approved by Zanjan University of Medical Sciences Ethics Committee (IR.ZUMS.REC.1398.430). Semen samples were obtained after informed consent from 40 infertile patients (20 asthenozoospermia and 20 teratozoospermia) referred to the Alzahra infertility clinic (Tabriz, Iran). Also, 20 fertile males who had fathered a child in the same clinic were enrolled as controls. Clinical characteristics data assessed for this study are summarized in Table 1. An experienced andrologist examined all subjects before enrollment into the study. The patients with genital tract obstruction (obstructive azoospermia), endocrine abnormalities (hypogonadism, diabetes, Hypothyroidism, and Hyperthyroidism), cryptorchidism, varicocele, leukocytospermia, oligozoospermia, abnormal testicle size, karyotype anomalies, Y chromosome microdeletions, and also patients with a history of alcohol consumption, drug abuse, and smoking were excluded.

**TABLE 1:** Spermatic parameters of participants

Characteristics	Asthenozoospermic participants	Teratozoospermic participants	Normozoospermic participants
Age (year)	34.5 ± 4	34.21 ± 7.47	33.06 ± 5.49
Volume (mL)	2.5 ± 0.62	3 ± 1.62	3.2 ± 1.38
Sperm concentration (×10^6^ mL^−1^)	59.75 ± 18.02	56 ± 19.57	70 ± 12.46
Total sperm count (×10^6^ per ejaculate)	165.5 ± 59.41	179.125 ± 116.78	213.75 ± 78.23
Progressive motility (%)	25 ± 6.25	40 ± 5.848	45 ± 10
Non‐progressive motility (%)	35 ± 5	35 ± 5	30 ± 5
Immotile spermatozoa (%)	35 ± 5	25 ± 10	22.5 ± 11.25
Normal morphology (%)	5	2	5 ± 1.25

*Note*: Data shown are mean ± SD for normal distributions, median (IQR) for non‐normal distributions.

Semen samples were collected by masturbation after a period of 3 to 5 days of sexual abstinence and were incubated at 37°C for a period of 30 to 60 minutes for liquefaction. Semen PH, volume, viscosity, sperm count, motility, and morphology were evaluated according to the protocol recommended by the World Health Organization (WHO) manual, fifth edition.^32^ Sperm concentration was defined using a Neubauer hemocytometer. Sperm motility was assessed by phase‐contrast microscopy at ×400 magnification. Sperm morphology assessment was carried out using Diff‐Quick stain. At least 200 spermatozoa were evaluated for each sample. The normozoospermic samples had normal sperm concentrations (15 × 10^6^/mL), normal sperm morphology ≥4%, and progressive motility (PR; ≥32%). In the asthenozoospermic group, less than 32% of the spermatozoa had progressive motility. The semen samples with less than 4% of normal sperm morphology were considered teratozoospermia. After semen analysis, the samples were centrifuged (2200 RPM, 10 minutes), then washed twice with Ham's F‐10 and centrifuged (300 RPM, 5 minutes) to separate spermatozoa and seminal plasma. The specimens were treated with somatic cell lysis buffer (SCLB; 0.1% sodium dodecyl sulfate, 0.5% Triton X; 100% in dH2O), then washed once with 1 mL phosphate‐buffered saline (PBS) to rule out the possibility of any somatic cell contamination. Then the microscopic inspection was used to verify the absence of somatic cells in the sperm suspension. The agarose gel electrophoresis was performed to ensure the absence of 18S and 28S ribosomal RNAs and somatic cell contaminants.

### 2.2 RNA extraction

Total RNA extraction was performed from purified sperm samples using the TRIzol method. Briefly, the sperm pellet was suspended in 1 mL of TRIzol reagent (RNA biotechnology company, IRAN) according to the manufacturer's instruction. The RNA pellet was then dissolved in 30 μL RNase‐free water. Nanodrop (Thermo Scientific, USA) was used to calculate the quality of the RNA samples (A260/280 ratio) and total RNA concentration.

### 2.3 Complementary DNA synthesis and qPCR

Complementary DNA (cDNA) was synthesized from 2 μg of total RNA using BioFACT 2X RT Pre‐Mix kit (Korea) in a final volume of 20 mL. The reaction was carried out at 25°C for 5 minutes, 50°C for 30 minutes, and 90°C for 5 minutes by Gradient thermocycler PCR (BIO‐RAD, Germany).

qPCR was carried out by SYBR Green Master Mix (Amplicon, Denmark) and Rotor‐Gene Q 6plex System (Qiagen, Germany) in a 20 mL total reaction volume, containing a specific primer set. qPCR was performed in three steps with the following program: for *SEPT4* and *GAPDH*: initial denaturing at 95°C for 15 minutes, 45 cycles of denaturation at 95°C for 15 seconds; primer annealing temperature at 60°C for 30 seconds; and elongation at 72°C for 20 seconds. And for *SEPT2*: 15 minutes at 95°C, 45 cycles of 15 seconds at 95°C and 30 seconds at 56°C, and 72°C for 20 seconds. Melting curves were obtained and analyzed after each run. The presence of one peak in melting curves and the single bands on agarose gel electrophoresis with appropriate size confirmed the specificity of the amplification. All qPCR reactions were performed in duplicate. The results were analyzed using the Rotor‐Gene Q Series Software 2.3.1 and were converted into threshold cycle values. *GAPDH* was used as the reference gene for data normalization. Table 2 summarizes the primer sequences and the resulting PCR product length.

**TABLE 2:** Primer sequences used for qPCR

Gene	Primer sequences (5′ to 3′ direction)	Amplified fragment size (bp)
SEPT2	F: CCATGCTCATCACCCACATGC	94
	R: CTGCCGCCTCTCTTGAGTCT	
SEPT4	F: ACCACAAGAAACGCAAAATCC	97
	R: TTGAAGTCCTCATCCTCATCAG	
GAPDH	F: AAGGTGAAGGTCGGAGTCAAC	102
	R: GGGGTCATTGATGGCAACAA	

### 2.4 Statistical analyses

The 2^−ΔΔCt^ method was used to compute the relative transcript levels of each gene. The Shapiro‐Wilk test was applied to check data normality. The data are presented as mean ± SD (SD) or median (interquartile range, IQR) for non‐normal distributions. The Mann‐Whitney two‐tailed test was used to statistically analyze differences of *SEPT2* and *SEPT4* transcript contents between groups. Spearman's correlation test (two‐tailed) was performed to assess any correlation between transcript content and sperm progressive motility. The statistical analyses of qPCR results were performed by GraphPad Prism 8. The statistical power analysis was calculated using G*Power 3.1.^33^ A value of *P* < .05 was considered statistically significant.

## 3 RESULTS

**FIGURE 2:**
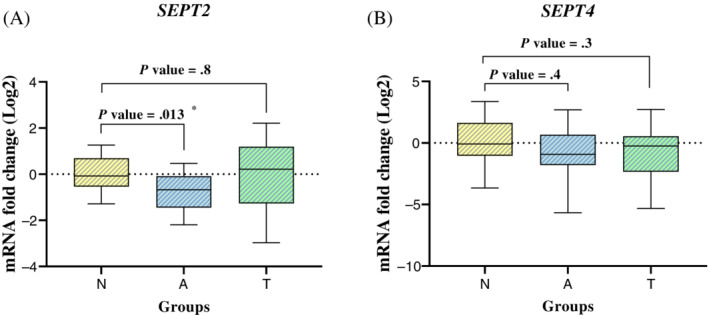
qPCR analysis of *SEPT2* (A) and *SEPT4* (B) genes in sperm samples of teratozoospermic, asthenozoospermic, and normozoospermic men. N, normozoospermic samples; A, asthenozoospermic samples; T, teratozoospermic samples

The transcript contents of *SEPT2* and *SEPT4* were determined in sperm samples using qPCR. *GAPDH* gene was used as an internal reference to normalize the expression of the genes. The transcript content of *SEPT2* was significantly decreased in the patients with asthenozoospermia compared with the normozoospermic individuals (*P* value = .013). Spearman's correlation analysis revealed a significant inverse correlation between *SEPT2* mRNA content and sperm progressive motility rate (r = −0.5441, *P* value = .013). The transcript content of the *SEPT2* was nearly identical between teratozoospermic and normozoospermic groups; therefore, no difference was observed between these two groups (*P* value = .8) (Figure 2A). Also, the *SEPT4* mRNA content was not significantly different in asthenozoospermic and teratozoospermic patients compared with the normozoospermic group (*P* value = .4, and *P* value = .3 respectively) (Figure 2B). The amplification efficiencies of *SEPT2*, *SEPT4*, and *GAPDH* were 1.907, 1.939, and 1.919, respectively. The power of the study was evaluated as 0.76.

## 4 DISCUSSION

The annulus connects the midpiece and principal piece of the mammalian sperm tail. The septins, a family of GTPase proteins, are the main components of the annulus.^23^, ^26^ SEPT2, 4, 6, 7, and 12 self‐assemble into an octameric complex, and these complexes become elongated by the end‐to‐end association, forming the ring structure of the septin in the annulus. SEPT2 and SEPT4 occupy the same position in this complex.^26^ The annulus is required for normal motility and morphology of the spermatozoa flagellum.^21^, ^22^ The investigation of transcript contents in spermatozoa is considered to be useful in identifying the pathogenic mechanisms involving in sperm motility and morphological defects.^13^, ^16^, ^18^, ^19^

In the current study, we evaluated *SEPT2* and *SEPT4* mRNA contents in asthenozoospermic, teratozoospermic, and normozoospermic samples. Our findings indicate that the mRNA content of *SEPT2* was significantly lower in patients with asthenozoospermia compared with the normozoospermic group. Although we observed the lower content of the *SEPT4* transcript in asthenozoospermic men compared with the control group, the difference was not significant. However, Li et al have revealed that SEPT4 mRNA and protein content was significantly decreased in sperm samples of idiopathic asthenozoospermia patients using RT‐PCR and western blot. Therefore, they introduced the decreased expression level of SEPT4 as a possible cause of idiopathic asthenozoospermia.^34^

The deletion of the *Sept4* gene in male mice resulted in infertility due to immotility and morphological defects of spermatozoa, including loss of the annulus, abnormal organization of the mitochondria, the disjunction of midpiece and principal piece, and abnormal bending of the flagellum forming a hairpin‐like structure.^21^, ^22^ Other septins, which are the main components of the annulus, were delocalized and failed to organize the annulus in the absence of the SEPT4. The annulus was replaced by a narrow segment, and the cortex was disorganized, leading to fragility and immotility of the spermatozoa. These findings indicate that the annulus is essential for cortical organization, mechanical integrity and motility of spermatozoa, and reproduction.^21^ Moreover, knockout of *Sept12* and *Tat1*, which are localized in the annulus, in male mice resulted in infertility, sperm immotility, defective or abnormal annulus, and bending of the sperm tail.^35^, ^36^ Also, disorganization or lack of the annulus was observed in humans with asthenozoospermia.^21^, ^25^, ^31^ Lhuillier et al reported the absence of TAT1, SEPT4, and SEPT7 proteins in one case with moderate asthenozoospermia lacking the annulus. Moreover, abnormal mitochondrial arrangement and disjunction between the midpiece and the principal piece were revealed, and peripheral doublets were absent in the axoneme. They considered the absence of annulus and flagellar disorganization to be associated with asthenozoospermia in the patient.^37^ Also, Hosseinifar et al identified a patient with moderate asthenozoospermia lacking the annulus and SEPT4 or SEPT7 proteins at the annulus in 75% of spermatozoa in the semen.^38^

In summary, our results in the teratozoospermic group indicated that the *SEPT2* and *SEPT4* transcript levels were not significantly different from the control group. We assume that our finding can be explained with one of two possibilities. First, there is no association between the *SEPT2* and *SEPT4* transcripts and teratozoospermia. Secondly, the mean percentage of morphologically normal spermatozoa in the control group was near the cutoff value determined by the WHO. No significant difference was observed for the *SEPT4* transcript between the asthenozoospermic and control groups. We observed that *SEPT2* was significantly differentially expressed between asthenozoospermic and normozoospermic men, which might be associated with poor motility. These observations might help in understanding the molecular mechanisms underlying asthenozoospermia. Further studies with a larger sample size are needed to confirm our findings and also investigate the association between the transcript contents of *SEPT2* and *SEPT4* with sperm morphological defects.

## FUNDING

The study was supported by Zanjan University of Medical Sciences (ZUMS) (Grant No. A‐12‐65‐19).

## CONFLICTS OF INTEREST

The authors declare that they have no conflict of interest.

## AUTHOR CONTRIBUTION

Conceptualization: Madiheh Mazaheri Moghaddam, Marziyeh Mazaheri Moghaddam.

Funding Acquisition: Alireza Biglari.

Investigation: Madiheh Mazaheri Moghaddam, Marziyeh Mazaheri Moghaddam, Mohammad Amini, Behzad Bahramzadeh, Amir Baghbanzadeh, Alireza Biglari, Ebrahim Sakhinia.

Project Administration: Alireza Biglari, Ebrahim Sakhinia.

Resources: Behzad Bahramzadeh, Ebrahim Sakhinia.

Supervision: Alireza Biglari, Ebrahim Sakhinia.

Validation: Madiheh Mazaheri Moghaddam, Marziyeh Mazaheri Moghaddam, Mohammad Amini, Behzad Bahramzadeh, Amir Baghbanzadeh, Alireza Biglari, Ebrahim Sakhinia.

Writing—Original Draft Preparation: Madiheh Mazaheri Moghaddam, Marziyeh Mazaheri Moghaddam, Amir Baghbanzadeh.

Writing—Review and Editing: Madiheh Mazaheri Moghaddam, Marziyeh Mazaheri Moghaddam, Mohammad Amini, Amir Baghbanzadeh, Alireza Biglari, Ebrahim Sakhinia.

All authors have read and approved the final version of the manuscript.

Madiheh Mazaheri Moghaddam and Alireza Biglari had full access to all of the data in this study and take complete responsibility for the integrity of the data and the accuracy of the data analysis.

## TRANSPARENCY STATEMENT

Madiheh Mazaheri Moghaddam affirms that this manuscript is an honest, accurate, and transparent account of the study being reported; that no important aspects of the study have been omitted; and that any discrepancies from the study as planned (and, if relevant, registered) have been explained.

## Data Availability

The authors confirm that the data supporting the findings of this study are available within the article.
